# Silvestrol exhibits significant *in vivo* and *in vitro* antileukemic activities and inhibits FLT3 and *miR-155* expressions in acute myeloid leukemia

**DOI:** 10.1186/1756-8722-6-21

**Published:** 2013-03-16

**Authors:** Houda Alachkar, Ramasamy Santhanam, Jason G Harb, David M Lucas, Joshua J Oaks, Christopher J Hickey, Li Pan, A Douglas Kinghorn, Michael A Caligiuri, Danilo Perrotti, John C Byrd, Ramiro Garzon, Michael R Grever, Guido Marcucci

**Affiliations:** 1 Division of Hematology, Department of Medicine, The Ohio State University, Columbus, OH, USA; 2 Department of Molecular Virology, Immunology and Cancer Genetics, The Ohio State University, Columbus, OH, USA; 3 Division of Medicinal Chemistry and Pharmacognosy, College of Pharmacy, The Ohio State University, Columbus, OH, USA; 4 Comprehensive Cancer Center, The Ohio State University, Columbus, OH, USA; 5 The Ohio State University Comprehensive Cancer Center, Biomedical Research Tower 460 W. 12th Ave, Columbus, OH, 43210, USA; 6 395 West Twelfth Avenue, Rm. 392 North Doan Office Tower, Columbus, OH, 43210, USA

## Abstract

**Background:**

Activating mutations [internal tandem duplication (ITD)] or overexpression of the FMS-like tyrosine kinase receptor-3 (*FLT3*) gene are associated with poor outcome in acute myeloid leukemia (AML) patients, underscoring the need for novel therapeutic approaches. The natural product silvestrol has potent antitumor activity in several malignancies, but its therapeutic impact on distinct molecular high-risk AML subsets remains to be fully investigated. We examined here the preclinical activity of silvestrol in *FLT3*-ITD and *FLT3* wild-type (wt) AML.

**Methods:**

Silvestrol *in vitro* anti-leukemic activity was examined by colorimetric cell viability assay, colony-forming and flow cytometry assays assessing growth inhibition and apoptosis, respectively. Pharmacological activity of silvestrol on *FLT3* mRNA translation, mRNA and protein expression was determined by RNA-immunoprecipitation, qRT-PCR and immunoblot analyses, respectively. Silvestrol *in vivo* efficacy was investigated using MV4-11 leukemia-engrafted mice.

**Results:**

Silvestrol shows antileukemia activity at nanomolar concentrations both in *FLT3*-wt overexpressing (THP-1) and *FLT3*-ITD (MV4-11) expressing AML cell lines (IC_50_ = 3.8 and 2.7 nM, respectively) and patients’ primary blasts [IC_50_ = ~12 nM (*FLT3*-wt) and ~5 nM (*FLT3*-ITD)]. Silvestrol increased apoptosis (~4fold, P = 0.0001), and inhibited colony-formation (100%, P < 0.0001) in primary blasts. Silvestrol efficiently inhibited *FLT3* translation reducing FLT3 protein expression by 80–90% and decreased *miR-155* levels (~60%), a frequently co-regulated onco-miR in *FLT3*-ITD-positive AML. The median survival of silvestrol-treated vs vehicle-treated mice was 63 vs 29 days post-engraftment, respectively (P < 0.0001).

**Conclusions:**

Silvestrol exhibits significant *in vivo* and *in vitro* antileukemic activities in AML through a novel mechanism resulting in inhibition of *FLT3* and *miR-155* expression. These encouraging results warrant a rapid translation of silvestrol for clinical testing in AML.

## Background

Acute myeloid leukemia (AML) is one of the most common types of leukemia. Today, only 40% of younger (age <60 years) and 10% of older (age ≥60 years) adults with AML treated with conventional chemotherapy achieve long-term survival [1]. The outcome for high-risk patients who are treated with allogeneic stem cell transplantation in first complete remission are encouraging, but lack of suitable donors, presence of co-morbidities and treatment-related toxicity and mortality has often limited the application of this approach. Therefore, novel therapeutic strategies that improve the currently poor outcome in AML patients while demonstrating an optimal toxicity index are highly needed.

Recurrent cytogenetics and molecular aberrations are known to impact the prognosis of AML. Importantly, several of these genomic aberrations may also constitute novel therapeutic targets. Gain-of-function mutations of the tyrosine kinase (TK) receptor encoding gene *FLT3* occur in approximately 30% of AML patients, and result in constitutive TK activity and, in turn, increasing growth and survival of leukemia blasts [2]. Of the *FLT3* mutations, the internal tandem duplication (*FLT3*-ITD) is associated with poorer outcome [3-8]. In addition, overexpression of the FLT3-wt receptor and its ligand (FL) occurs in a high percentage of AML and the subsequent autocrine stimulatory loop may contribute to the pathogenesis and aggressiveness of the disease [9,10]. Therefore, investigating compounds that can inhibit both mutant and overexpressed wild type FLT3 in AML leukemia is warranted.

Smith et al. demonstrated that *FLT3*-ITD likely constitutes a driver mutation in AML and therefore it may represent not only as a prognosticator but also a potential therapeutic target [11]. Emerging small molecule inhibitor compounds have been shown to interfere with the aberrant FLT3 TK activity and lead to arrest of leukemia growth [6,12]. Unfortunately the clinical impact of these compounds as single agents or in combination with chemotherapy has not yet fulfilled the promise, likely because of their relatively low potency, lack of specificity and the early onset of mechanisms of resistance [13-15]. This underscores the need for additional strategies that effectively target aberrant FLT3 activity in AML blasts and improve the currently poor survival rate in high-risk patients with *FLT3*-driven AML.

Silvestrol is a cyclopenta[*b*]benzofuran rocaglate with a unique dioxanyl ring-containing side chain [16,17]. It was isolated from the Indonesian plant *Aglaia foveolata*, structurally characterized, and tested for anti-tumor efficacy [16]. Silvestrol showed activity against several solid tumor cell lines [18-21] as well as primary chronic lymphocytic leukemia cells at nanomolar concentrations, and prolonged survival in a murine model of B cell acute lymphoblastic leukemia [22]. More recently, silvestrol was reported to have synergistic activity against AML cell lines when combined with chemotherapy [23]. Pelletier et al., demonstrated that silvestrol interferes with assembly of the eIF4F translation complex by promoting an aberrant interaction between capped mRNA and eIF4A, thus blocking protein synthesis at the initiation step [21,24]. This inhibition of protein synthesis results in a preferential depletion of proteins with short half-lives, such as MCL1, to which leukemia and cancer cells may be addicted and thrive on [22,24]. Therefore, we hypothesize that silvestrol could also inhibit translation of *FLT3* mRNA and in turn downregulate the expression of FLT3 and decrease aberrant tyrosine kinase in *FLT3*-driven AML. Thus, we sought to examine the *in vitro* and *in vivo* anti-leukemic and biological activity of silvestrol in *FLT3*-ITD or *FLT3*-wt overexpressing AML cell lines and primary blasts.

## Methods

### Reagents

Silvestrol was kindly provided by Dr. A. Douglas Kinghorn. PKC412 was purchased from LC Laboratories (Woburn, MA, USA).

### Cell lines and primary blasts

MV4-11 and THP-1 cells (ATCC, Manassas, VA) were cultured in RPMI 1640 medium supplemented with 10% calf serum. Blasts from AML patients were maintained in RPMI 1640 medium supplemented with 30% fetal bovine serum, 1% HEPES buffer, and 1× StemSpan CC100 (StemCell Technologies, Vancouver, BC, Canada) containing IL-3, IL-6, FLT3 ligand and SCF. All cells were incubated at 37°C with 5% CO_2_. Patient AML blasts were obtained from apheresis blood samples collected from patients treated at the Ohio State University (OSU) and stored in the OSU Leukemia Tissue Bank. Informed consent to use cells for investigational studies was obtained from each patient under an OSU Institutional Review Board-approved protocol, according to the Declaration of Helsinki. Authenticating tests of these cell lines was done using monoclonal antibodies and *FLT3* mutational analysis.

### Immunoblot analyses

Cells were suspended 30 min in 1 × lysis buffer (20 mM Hepes, 150 mM NaCl, 0.1% NP40) containing protease inhibitor cocktail III (Calbiochem, Darmstadt, Germany) and lysate was recovered by centrifugation. Lysates were separated using 4-20% SDS-PAGE and transferred to PVDF membrane (GE Healthcare, Piscataway, NJ). Membranes were blocked using 5% milk or BSA in 1 × TBS with 0.1% Tween 20 (1 × TBS-T) for 1 hour at room temperature with shaking, then incubated overnight at 4°C in the following primary antibodies diluted in 1 × TBS-T with 5% milk or BSA: actin (Santa Cruz Biotechnology, Santa Cruz CA), FLT3 (Cell Signaling, Danvers, MA), phosphorylated and total STAT5 (Cell Signaling), P65 antibody (Billerica, MA). Membranes were washed using 1 × TBS-T, incubated with HRP-conjugated secondary antibodies diluted in 1 × TBS-T with 5% milk or BSA, washed, and developed using ECL Western Blotting Detection reagents (GE Heathcare).

### RNA immunoprecipitation (RIP), RNA extraction, Real-Time RT-PCR

MV4-11 cells were treated with 50 nM silvestrol for 3 hour, lysed (5 min) in 100 mM KCl, 5 mM MgCl_2_, 10 mM HEPES [pH 7.0], 0.5% NP-40, 1 mM dithiothreitol (DTT), 100 units/ml RNase OUT (Invitrogen), 400 mM vanadyl-ribonucleoside complex and protease inhibitors (Roche, Mannheim. Germany). Extracts were clarified and stored at −80°C. Anti-eIF4E antibody (cell signaling) and goat IgG (Sigma, St. Louis, MO) were incubated with protein sepharose A/agarose G-coupled beads overnight. Beads were subsequently washed four times with 50 mM TRIS/HCl, pH 7.0, 150 mM NaCl, 1 mM MgCl_2_, and 0.05% NP-40, and twice after addition of 1 M urea. Precipitates were digested with proteinase K (55°C), and eIF4E-associated mRNAs were isolated using Trizol reagent (Invitrogen, Grand Island, NY). cDNA was synthesized using SuperScript III reagents (Invitrogen) and the TaqMan MicroRNA Reverse Transcription Kit (Applied Biosystems, Foster City, CA) according to the manufacturer’s instructions. Quantitative Real-Time RT-PCR for *FLT3* and *PU.1* genes and *miR-155* and *miR-34a* expression was performed using commercially available TaqMan Gene Expression Assay primers and probes and the 7900HT Fast Real-Time PCR System (Applied Biosystems). The comparative cycle threshold (C_T_) method was used to determine the expression levels normalized by the internal control *18S* for gene expression.

### Clonogenic and viability analysis

Methylcellulose clonogenic assays were carried out by plating 2 × 10^4^ primary blasts in 0.9% MethoCult (Stem Cell Technologies). Colonies (>100 mm) from cell lines and primary cells were scored 14 days later. Growth inhibition assays were performed. Briefly, 5.0 × 10^4^ cells were incubated in triplicate in a 96-well plate in the presence or absence of the different concentrations of silvestrol in a final volume of 100 μl for 24, 48 and 72 hours at 37°C. Thereafter, 20 μl of the CellTiter 96® AQ_ueous_. One Solution Reagent which contains tetrazolium compound [3-(4,5-dimethyl-2-yl)-5-(3-carboxymethoxyphenyl)-2-(4-sulfophenyl)-2H-tetrazolium, inner salt; MTS] and an electron coupling reagent (phenazine ethosulfate; PES) (Promega, Madison WI) was added to each well. After 4 hours incubation at 37°C, the optical density at 490 nm was measured. Cell viability was calculated with respect to the control samples. At least three independent experiments were performed.

### Flow cytometry

For FLT3 detection, cells (5 × 10^5^) were washed with phosphate-buffered saline (PBS) and resuspended in 50 μl binding buffer containing 5 μL FLT3 antibody (BD Biosciences, Billerica, MA). After 15 min incubation, cells were washed with PBS, resuspended in 400 μL flow buffer and analyzed on a FACSCalibur cytometer (BD Biosciences). To assess apoptosis, AML cells were incubated with 10, 30 and 50 nM silvestrol for 24 hours. Cells (5 × 10^5^) were then washed with PBS and resuspended in 50 μl binding buffer containing 2 μL of annexin V-FITC stock (BioWhittaker, Inc, Walkersville, MD) and 5 μL propidium iodide (PI) (BD Biosciences). After 20 min incubation, fluorescence was quantified by flow cytometry on a FACSCalibur instrument.

### MV4-11 xenograft murine model

This model was developed recently in our laboratory and described previously [25]. Briefly, 4 ~ 6 week-old non-obese diabetic severe combined immunodeficient gamma (NSG) mice (NOD.Cg-Prkdcscid Il2rgtm1Wjl/SzJ, The Jackson Laboratory, Bar Harbor, ME) were intravenously (*i.v*) injected via tail vein with 2 × 10^7^ MV4-11 cells. Two months later, the spleen mononuclear cells (MNCs) were isolated from MV4-11-injected mice (1^st^-adapted MV4-11 cells in NSG mice). The adapted spleen MNCs were injected into a new cohort of NSG mice via tail vein. The loading dose of cells was reduced to 50% in this second transplantation. About a month later, the spleen MNC were isolated and a 3^rd^ transplantation was performed using 0.5 × 10^7^ cells/mouse spleen MNC from 2^nd^ adapted NSG mice. The sequential transplants were performed using 0.5 × 10^6^ cells/mouse spleen MNC from 3^rd^ adapted NSG mice. This provides a more aggressive and fast onset of AML-like disease, thereby allows for rapid read-out of the experiment. Indeed, we observed development of leukemia at only two weeks from injection and a median survival of approximately 4 weeks after engraftment of the adapted MV4-11 cells from the 3^rd^ transplant. The complete blood count and FACS analysis of CD45 and FLT3 expression and cytospin were examined weekly to monitor the progression of disease. All the experiments were conducted in accordance with the institutional guidelines for animal care and use.

Spleen cells (0.5 × 10^6^) from MV4-11 transplanted NSG mice were intravenously (iv) injected into NSG mice via tail vein, and divided into groups for vehicle (hydroxypropyl beta-cyclodextrin in 30% sterile water; N = 6) or silvestrol (1.5 mg/kg in vehicle; N = 10) treatment. One control mouse (no leukemia/no treatment) was also included. Two to three weeks after engraftment, white blood count (WBC) and FLT3 expression by flow cytometry were assessed to confirm transplantation. Treatments with silvestrol or vehicle were initiated 21 days after engraftment (based on disease signs documented by WBC count and FLT3 expression). Administration was by intraperitoneal injection every 48 hours for up to three weeks or until euthanasia criteria were met. Expected median survival of untreated animals in this model is 28 days. Mice were weighed daily and checked for signs of dehydration, discomfort or toxicity. On day of administration, doses were recalculated for each animal after weighing to maintain 1.5 mg/kg. For the pharmacodynamic study, 6 mice were used (3 per group, vehicle and silvestrol). These mice were given 3 doses of either vehicle or silvestrol; and 48 hours following the third dose, spleens were isolated and mononuclear cells obtained for immunoblotting assay. For pathological examination, tissue sections from the liver, spleen and bone marrow sternum were fixed on formalin, embedded in paraffin blocks, and H&E stained.

### Wright-Giemsa staining

Morphological signs of apoptosis were detected by Wright-Giemsa staining. Smears of control and treated cells were stained with Wright-Giemsa solution for 25 min, rinsed with distilled water and air dried. Cell morphology was studied by light microscopy.

## Results

### Silvestrol antileukemia activity in AML cells

**Figure 1:**
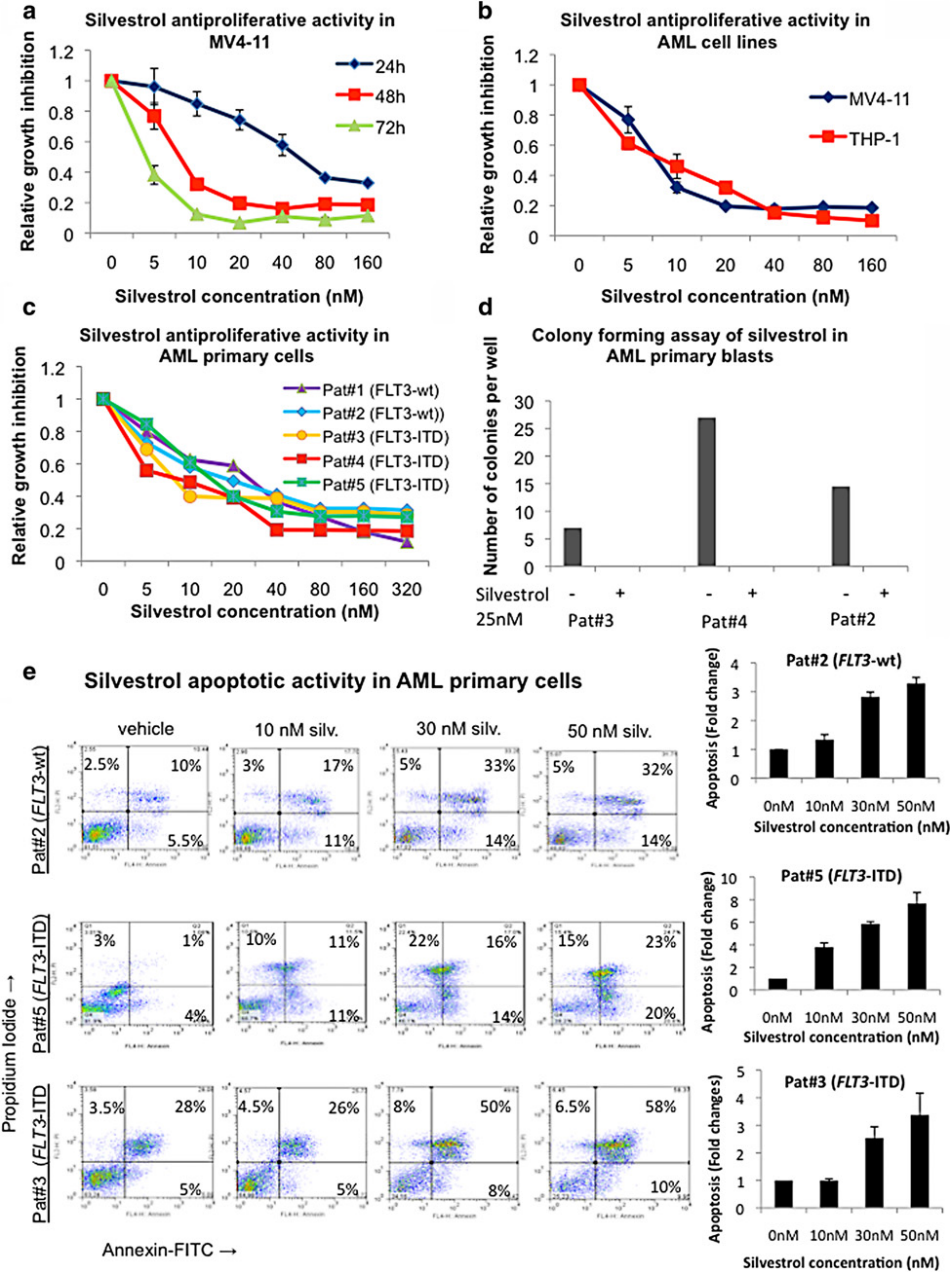
**Antileukemic activity of silvestrol *in vitro:*** (**a**) MV4-11 cells were incubated with 5 to 160 nM silvestrol. Cell viability was evaluated by a MTS assay at 24, 48 and 72 hours. (**b**) AML cell lines were incubated with 5 to 160 nM silvestrol, and viability was evaluated by a MTS assay 48 hours later. (**c**) AML primary cells unmutated *FLT3* or with *FLT3-ITD* were incubated with 5 to 320 nM silvestrol. Cell viability was evaluated by MTS assay 48 hours following treatment. (**d**) Colony forming ability in primary blasts from *FLT3-ITD* positive and negative AML patients treated with 25 nM of silvestrol and scored 14 days later. (**e**) Primary blasts from *FLT3-ITD* positive and negative AML patients treated with 10, 30 and 50 nM of silvestrol for 48 hours and analyzed by annexin/PI flow cytometry. Values are presented as mean ± SEM.

The antileukemia activity of silvestrol was first tested in AML cell lines. MV4-11 cells are *FLT3*-ITD positive, whereas THP-1 cells are negative for *FLT3*-ITD but express robust levels of *FLT3-*wt. Silvestrol impacted leukemia growth (measured by MTS) in a dose- (5 to 160 nM) and time-dependent manner (Figure 1a). At 48 hours, IC_50_ values (concentration required to inhibit growth to 50% of control) were 2.7 and 3.8 nM in MV4-11 and THP-1 cells, respectively (Figure 1b and Table 1). Primary blasts from three AML patients with *FLT3*-ITD and two AML patients with *FLT3-*wt were treated with 5 to 320 nM silvestrol. A dose-dependent decrease in proliferation was observed, with IC_50_ values at 48 hours of 3.7, 4.9, and 6.4 nM for the blasts from the *FLT3*-ITD patients (n = 3), 16.9 and 6.6 nM for the blasts from the *FLT3*-wt patients (n = 2; Figure 1c and Table 1). We also performed a colony forming assay of silvestrol in primary samples from three of the five patients, including two of the *FLT3*-ITD positive cases. We observed no colony formation for any of the tested samples when blasts were treated with 25 nM of silvestrol (Figure 1d).

**Table 1:** **Calculated IC**_**50 **_**values for AML cell lines and primary blasts at 48 h**

**Cell line**	**FLT3 status**	**IC**_**50 **_**(95% Confidence intervals)**
MV4-11	*FLT3*-ITD	2.65 nM (1.49 to 4.72)
THP-1	*FLT3*-wt	3.81 nM (2.46 to 5.09)
**Primary blasts**		
Pat #1	*FLT3*-wt	16.9 nM (13.6 to 21.1)
Pat #2	*FLT3*-wt	6.61 nM (5.67 to 7.70)
Pat #3	*FLT3*-ITD	3.65 nM (2.21 to 6.04)
Pat #4	*FLT3*-ITD	4.89 nM (3.90 to 6.12)
Pat #5	*FLT3*-ITD	6.41 nM (4.07 to 10.1)

To determine whether exposure to silvestrol resulted in apoptosis, we utilized annexin and PI staining of cells treated with 10–50 nM silvestrol for 48 hours. A dose-dependent increase in apoptosis was observed in silvestrol treated compared with vehicle-treated primary blasts from *FLT3-*ITD (n = 3) as well as *FLT3*-wt (n = 2) AML patients (Figure 1e). In AML primary cells, silvestrol induced 1 to 7.5 fold increase in apoptosis in both *FLT3*-wt and *FLT3*-ITD blasts (P = 0.0001 for every tested case) treated with 10, 30 and 50 nM of silvestrol compared with vehicle treated controls.

### Silvestrol downregulates FLT3 expression through inhibition of *FLT3* translation initiation

Silvestrol interferes with assembly of the eIF4F translation complex by promoting an aberrant interaction between capped mRNA and eIF4A, thus blocking protein synthesis at the initiation step [21,24]. This inhibition of protein synthesis results in a preferential depletion of proteins with short half-lives to which leukemia and cancer cells may be addicted [22,24]. However, whether silvestrol also inhibits *FLT3* initiation of translation resulting in inhibition of FLT3 protein synthesis has not been reported. Thus, using an RNA immunoprecipitation assay, we tested this possibility.

**Figure 2:**
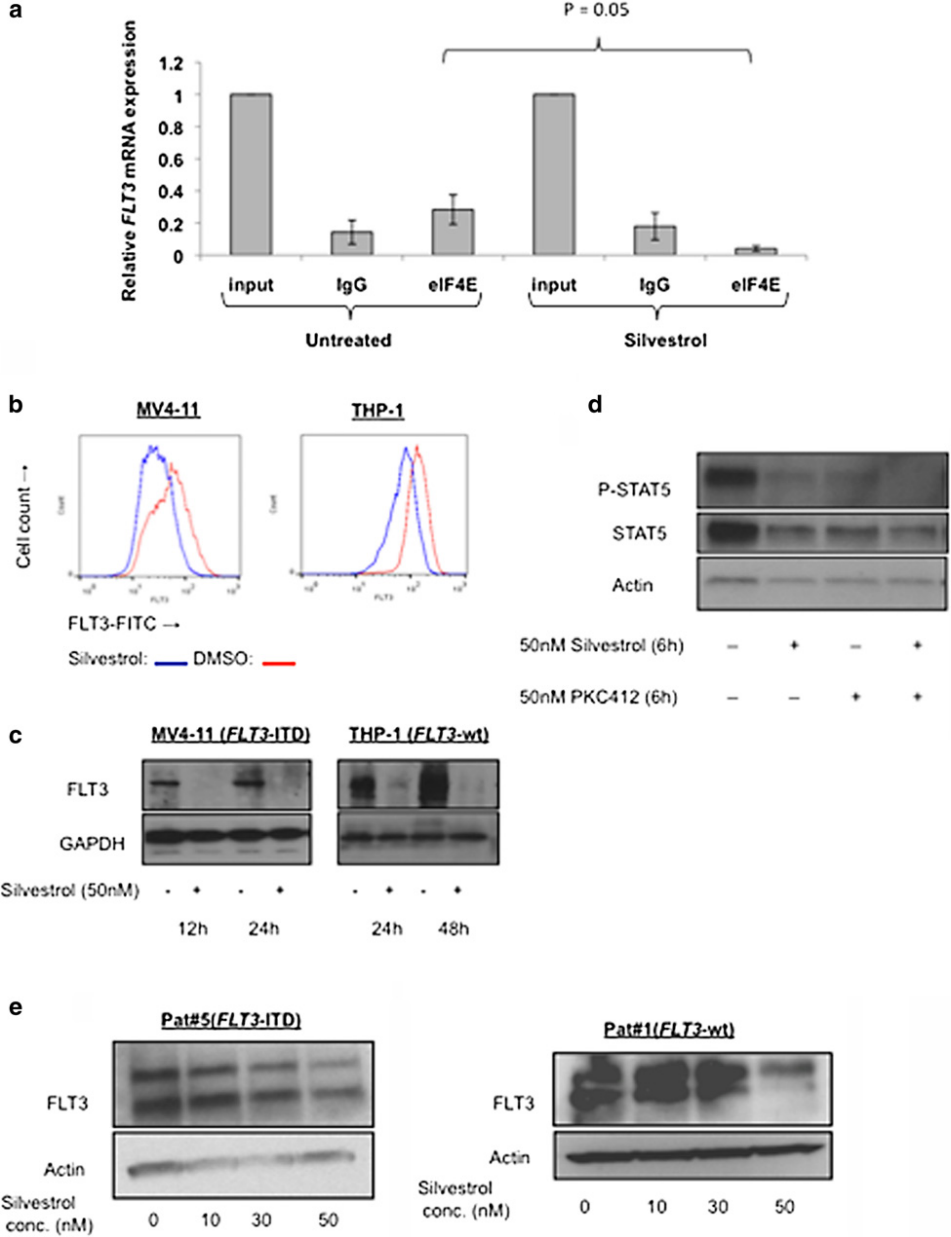
**Silvestrol down-regulates FLT3:** (**a**) *FLT3* mRNA expression in eIF4E immunoprecipitates from 50 nM silvestrol treated MV4-11 cells compared with that from untreated cells. Values are presented as mean ± SEM. (**b**) Expression of FLT3 examined by flow cytometry in MV4-11 and THP-1 cells after 24 hours exposure to 50 nM silvestrol. Blue = silvestrol-treated samples; Red = vehicle-treated control. (**c**) AML cells treated with 50 nM silvestrol and examined for FLT3 protein expression by immunoblotting. (**d**) MV4-11 cells treated with silvestrol or PKC412 (50 nM each) for 6 hours, and assessed for STAT5 phosphorylation by immunoblotting. (**e**) Primary AML cells incubated with increasing concentrations of silvestrol for 24 hours, and assessed for FLT3 protein expression by immunoblotting.

Because of the difficulty immunoprecipitating eIF4A protein for the RNA immunoprecipitation assay using commercially available antibodies, we utilized an antibody against eIF4E, a subunit of the initiation of translation complex that binds the mRNA cap structure. To avoid the interference due to cell death usually observed with more prolonged drug exposure and at the same time to ensure adequate pharmacologic activity, cells were exposed to silvestrol at concentrations that were higher than the calculated *in vitro* IC_50_ but that were also achievable *in vivo*, and harvested at 24 hours or earlier time points [26]. MV4-11 cells were treated with 50 nM silvestrol for 3 hours and eIF4E was immunoprecipitated in three different experiments. Associated RNA was then assessed for depletion of *FLT3* RNA by quantitative RT-PCR. While no change in *FLT3* mRNA levels was observed in total RNA from MV4-11 cells treated with silvestrol compared with vehicle-treated cells, a five-fold depletion of *FLT3* mRNA was detected in eIF4E immunoprecipitates from silvestrol-treated cells compared with vehicle-treated cells at 3 hours (Figure 2a). This resulted in 54% and 46% reduction in FLT3 receptor expression (by flow cytometry) (Figure 2b) and 92% and 84% reduction in FLT3-ITD or FLT3-wt protein levels (by western blot) respectively in MV4-11 and THP-1 cells, exposed to 50 nM silvestrol for 24 hours, compared with vehicle-treated controls (Figure 2c). Since total FLT3 protein levels were almost undetectable following silvestrol treatment, phosphorylated FLT3 was expected with be depleted as well. Therefore, TK activity was determined by measuring phosphorylation of the FLT3 target protein STAT5 in MV4-11 cells, which was found decreased following silvestrol treatment (Figure 2d). Similarly, down-regulation of FLT3 protein was observed in silvestrol-treated primary blasts (*FLT3*-wt and *FLT3*-ITD) (Figure 2e).

### Silvestrol downregulates *miR-155* expression in *FLT3*-ITD positive AML

**Figure 3:**
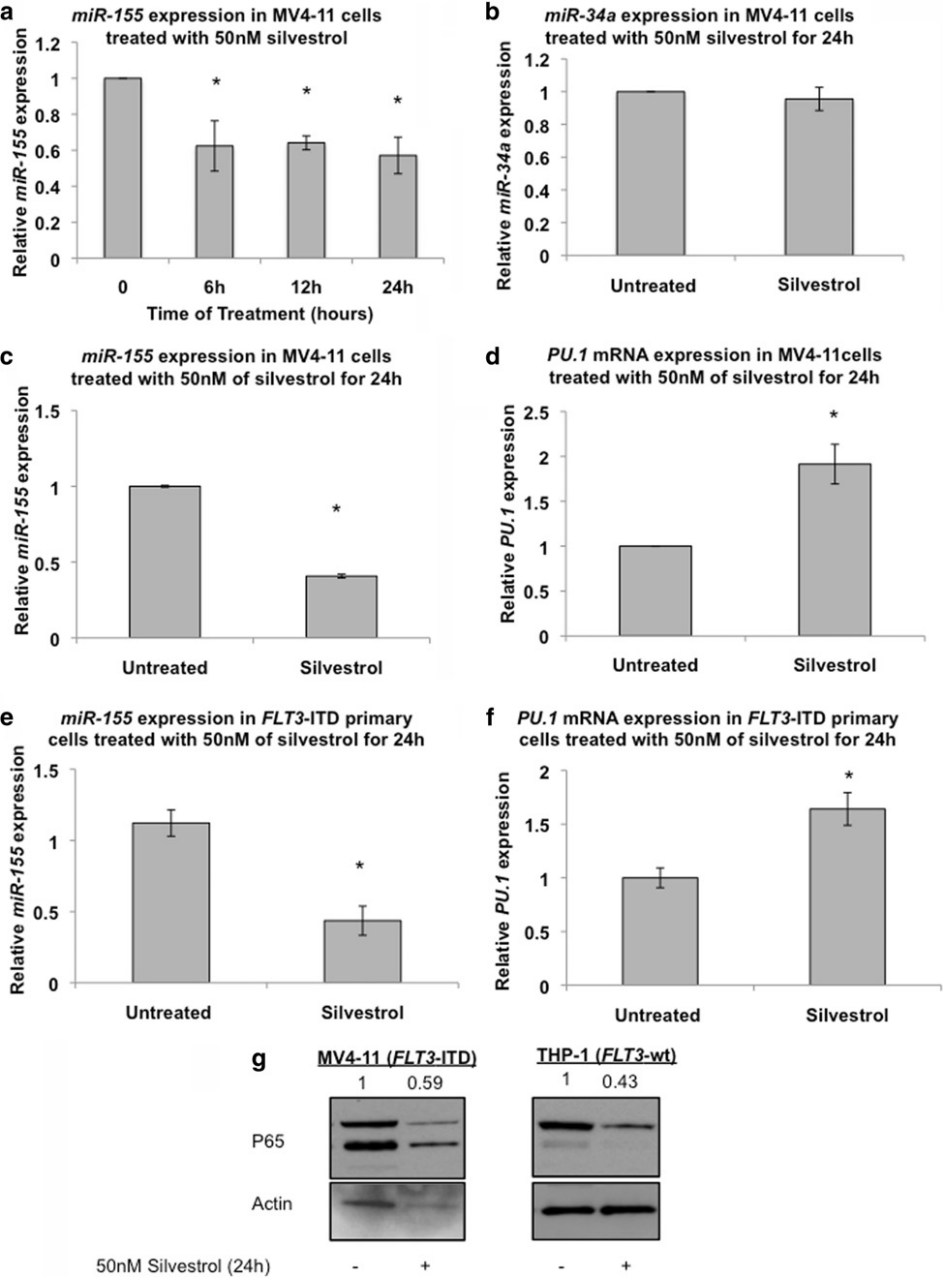
**Silvestrol down-regulates *miR-155 *and upregulates *miR-155 *target *PU.1*:** (**a**) MV4-11 cells were treated with 50 nM of silvestrol and *miR-155* expression was measured and normalized to *U44* expression at 6, 12 and 24 hours following silvestrol treatment. (**b**) MV4-11 cells were treated with 50 nM of silvestrol for 24 hours, and *miR-34a* expression was measured thereafter. (c,d) MV4-11 cells were treated with 50 nM of silvestrol for 24 hours, and *miR-155* (**c**) and *PU.1* expression (**d**) levels were measured thereafter. (e,f) *FLT3*-ITD positive primary cells were treated with 50 nM of silvestrol for 24 hours, and *miR-155* expression (**e**) and *PU.1* expression (**f**) levels were measured thereafter. (**g**) MV4-11 and THP-1 cells were treated with 50 nM of silvestrol for 24 hours and assessed for P65 protein expression by immunoblotting. Values are presented as mean ± SEM.

MicroRNAs are short, non-coding RNA that disrupt translation of mRNA targets, thereby resulting in down regulation of the mRNA corresponding encoded proteins. *miR-155* has a known oncogenic activity in hematologic malignancies [27,28]. Sustained expression of this miR in hematopoietic stem cells causes a myeloproliferative disorder [29]. We and others reported that *miR-155* is up-regulated in *FLT3*-ITD positive AML compared with *FLT3*-wt AML, although whether this microRNA directly contributes to the leukemogeneic activity of *FLT3*-ITD is unknown [4,30,31]. Having shown that silvestrol downregulated FLT3-ITD protein, next we tested whether silvestrol also altered the expression of *miR-155* that appears co-regulated with *FLT3*. Thus, *miR-155* expression was measured in MV4-11 cells treated with 50 nM of silvestrol by qRT-PCR. We found approximately a 60% decrease in *miR-155* expression in silvestrol treated compared with untreated cells (P = 0.05), while no significant change was observed in the expression of an unrelated miRs (i.e., *miR-34*) (Figure 3a, b and c). In order to examine the effect of silvestrol on *miR-155* function, we treated MV4-11 cells with 50 nM silvestrol and assessed the mRNA expression of the *miR-155* target gene *PU.1*[32]. Twenty-four hours following treatment with silvestrol, *PU.1* expression showed about 2 folds increase in the mRNA expression level compared with untreated MV4-11 (P = 0.04) (Figure 3d). Similar results were obtained in *FLT3*-ITD positive primary blasts; these cells exhibited 60% decrease in *miR-155* expression (P < 0.01) and a 1.6-fold increase in *PU.1* expression (P < 0.01), 24 hours following treatment with 50 nM of silvestrol compared with untreated cells (Figure 3e and f).

The mechanisms through which constitutively activated *FLT3* associates with increased *miR-155* expression are unknown. However, *miR-155* has been reported to be a target of NF-κB, which is also potentially activated in AML blasts with constitutively activated *FLT3*[33,34]. Additionally, Gerlof et al. reported that *FLT3*-ITD induces the oncogenic *miR-155* via STAT5 and NF-κB (p65) [35]. Given that silvestrol belongs to the rocaglate derivatives, which have been reported to inhibit the activity of NF-κB [18,36], we then verified the effect of silvestrol on NF-κB protein levels. When we treated MV4-11 and THP-1 cells with 50 nM of silvestrol for 24 hours, we observed decrease in NF-κB (P65) protein expression in silvestrol-treated cells compared with vehicle-treated cells (Figure 3g). This suggests that silvestrol may induce *miR-155* downregulation likely through direct inhibition of NF-κB (*FLT3*-ITD-negative THP-1 cells), and indirect inhibition of NF-κB via *FLT3*-ITD-dependent mechanisms (*FLT3*-ITD-positive MV4-11 cells). Nevertheless, the compound effectively targets both the microRNA and the mutant protein that may concurrently contribute to the aggressiveness of *FLT3*-ITD AML.

### Activity of silvestrol in *FLT3*-ITD positive leukemia grafts

**Figure 4:**
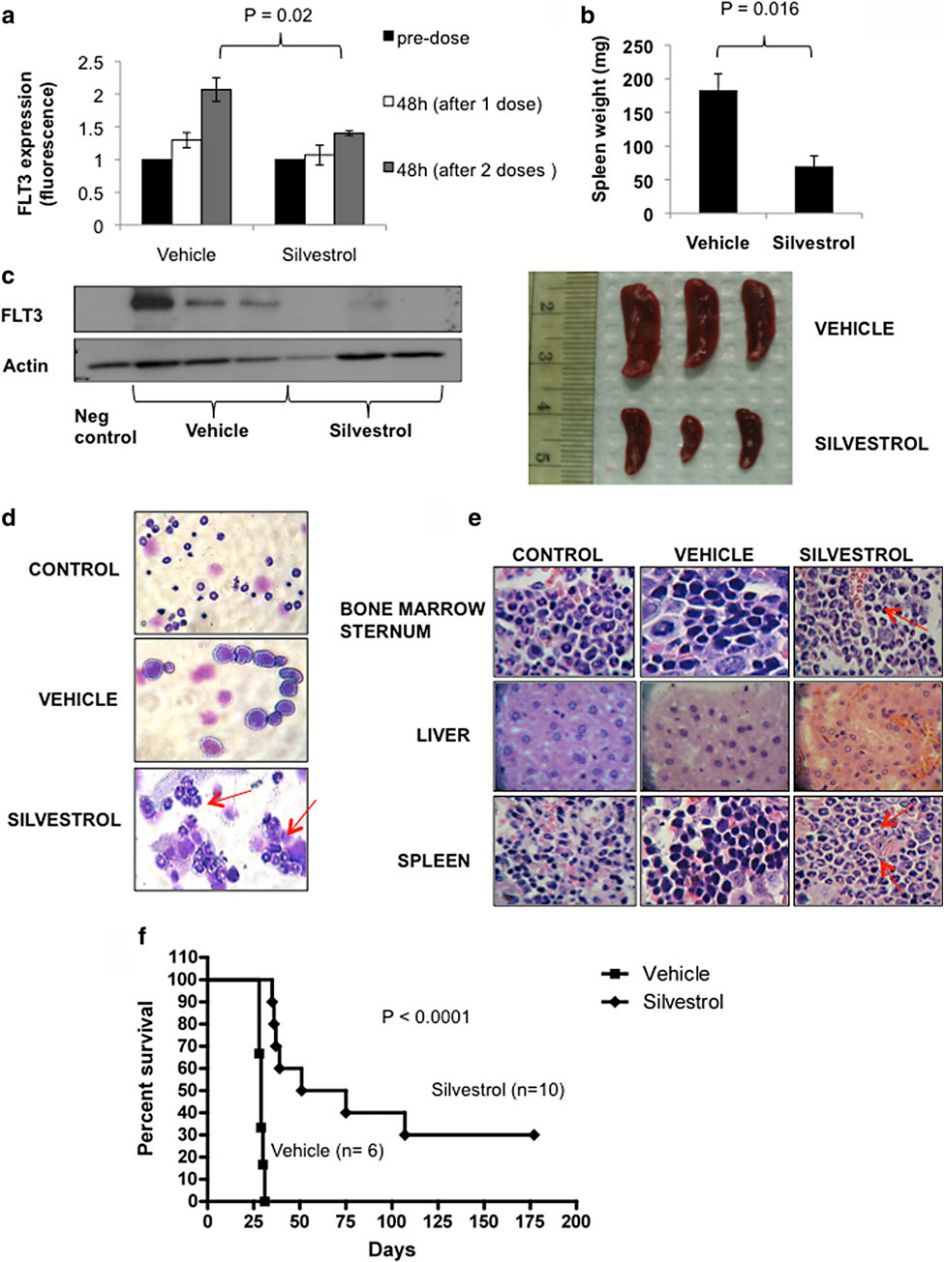
***In vivo *antileukemic activity of silvestrol in *FLT3*-ITD positive MV4-11 xenograft leukemia mouse model:** (**a**) FLT3 expression by flow cytometry evaluated 48 hours after 1^st^ and 2^nd^ doses. (**b**) Spleen size after 3 silvestrol doses (day 6 of treatment) (**c**) Immunoblot of FLT3 protein expression in spleens from silvestrol-treated mice and vehicle-treated controls. (**d**) Cytospins of bone marrow cells and (**e**) histopathology of bone marrow (sternum), spleen, and liver from the silvestrol-treated and vehicle-treated leukemic mice and age-matched (non-leukemic) control mice showing the infiltration of leukemic blasts in the vehicle treated and signs of differentiation in the silvestrol treated mice (see red pointing arrows). Stain: H&E; magnifications, 400×. (**f**) Survival analysis of silvestrol-treated leukemic mice (N = 10) compared with the vehicle-treated controls (N = 6). Values are presented as mean ± SEM.

A MV4-11 leukemia graft murine model was employed to investigate the *in vivo* efficacy of silvestrol in AML. NSG mice were subjected to secondary transplant with MV4-11 cells harvested from the spleen of previous MV4-11 engrafted mice that developed an aggressive AML-like disease. Twenty-one days post-engraftment, mice were treated intraperitoneally with vehicle (Group 1) or 1.5 mg/kg silvestrol (Group 2) every other day for 3 weeks. The silvestrol dose and schedule used here were previously reported for lymphoid leukemia models [22,37]. Forty-eight hours following the first two doses, blood samples with circulating MV4-11 cells were taken from 3 mice from each group, and FLT3 expression was assessed by flow cytometry. A two- and three-folds increase in FLT3 expression was observed in vehicle-treated mice 48 hours after the first (P = 0.07) and second (P = 0.024) treatment doses compared with pretreatment baseline, while no significant change was found in the silvestrol treatment group (Figure 4a). After 3 doses (day 6 of treatment), spleens from three mice from each group were examined. Spleens from silvestrol treated mice were 60% smaller (P = 0.016) (Figure 4b), and showed an 80% reduction in FLT3 protein (P = 0.002) compared with vehicle-treated controls (Figure 4c). Cytospins of bone marrow cells and histopathology of bone marrow (sternum), spleen, and liver sections from MV4-11-engrafted mice treated with vehicle showed extensive infiltration of blast cells. In contrast, cytospins and histopathology from silvestrol-treated leukemic mice were similar to those of the age-matched control mice (Figure 4d, e). Silvestrol-treated leukemic mice (n = 10) survived significantly longer than the vehicle-treated controls (n = 6) (median survival: 63 days from engraftment vs. 29 days, respectively; P < 0.0001). All mice in the vehicle group died by day 31 from engraftment (day 11 from treatment start), while 50% of silvestrol-treated mice were still alive on day 74 from engraftment (day 54 from treatment start) (Figure 4f). Of the silvestrol-treated mice, 30% were still alive at 6 months from experiment start day. When sacrificed, these survived mice showed no signs of leukemia.

## Discussion

The natural product silvestrol has been shown to inhibit translation initiation by modulating the interaction of capped mRNA with the RNA helicase eIF4A [24]. Proto-oncogenes such as *CCND1*, *MCL1*, and *MYC* tend to encode proteins with short half-lives, and are therefore more dependent on active translation to maintain protein levels that support malignant cell growth and survival. Such factors and in turn cancer cells addicted to them, are thus expected to be more sensitive to translation inhibition by silvestrol, as was recently reported [21-23,37]. To our knowledge, the effect of silvestrol on FLT3 expression has not been previously reported. Here we report that silvestrol also inhibits *FLT3* mRNA translation thereby causing depletion of the encoded oncoprotein, inhibition of the protein’s aberrant tyrosine kinase activity. Silvestrol caused growth arrest and apoptosis in AML cell lines and primary blasts treated at nanomolar concentrations. These results therefore extend the list of the oncoproteins targeted by silvestrol and support the antileukemia activity of this compound.

Most importantly, we showed a remarkable *in vivo* activity of silvestrol in a *FLT3*-ITD leukemia engraftment model. Mice engrafted with adapted MV4-11 cells developed leukemia and had a median survival of only 4 weeks. Treatment with silvestrol had no obvious toxicity and lowered the FLT3 expression to undetectable levels after administration of only three doses. Silvestrol treatment significantly prolonged survival and even cured approximately 30% of the animals, further reinforcing the concept that pursuing suppression of the FLT3-ITD protein expression may be a valid therapeutic approach in *FLT3*-driven AML. Recently, there has been an increased interest in utilizing small molecule kinase inhibitors to target aberrant TK activity of FLT3 mutants that contribute to leukemia growth and poor outcome in AML patients [38]. Unfortunately, the lack of selectivity, inadequate pharmacokinetics, and early onset of resistance resulted in relatively disappointing outcomes with these agents [39,40]. Nevertheless, FLT3 down-regulation via siRNA has been shown to result in growth inhibition and apoptosis in *FLT3*-ITD-positive AML cells [33,41]. This implicates that interfering not only with aberrant activity of this kinase but also with FLT3 expression might be a useful therapeutic strategy in AML. Thus, drugs like silvestrol, that have the ability to decrease the expression of FLT3 may be clinically useful in this distinct subset of AML addicted to the FLT3 aberrant TK activity.

Although activity of silvestrol on the FLT3 expression was impressive, it is likely that silvestrol as a natural product and an inhibitor of eIF4A has the ability to alter the expression and activity of several other targets that may contribute to the impairment of leukemia growth. Nevertheless, the antileukemia activity of this natural product may result favorable for those *FLT3*-driven molecular subsets of AML that are resistant to chemotherapy and/or enzymatic kinase inhibitors. To this end, we not only showed silvestrol-related FLT3 suppression, but also downregulation of *miR-155*, an oncomiR whose expression is likely regulated by NF-κB often found constitutively activated in *FLT3*-driven AML [42,43]. This microRNA has been shown to be upregulated in *FLT3*-ITD positive AML, and is thought to contribute to leukemia growth and aggressiveness in these molecular subsets of AML through downregulation of several targets including *PU.1*[4,31,44]. Indeed, we showed that silvestrol increased *PU.1* expression, which has been previously reported to inversely correlate with *FLT3* levels [45]. Thus, the dual activity of silvestrol on FLT3 and *miR-155* expression suggests that this compound may represent a potentially valuable therapeutic approach to this high-risk AML subset.

## Conclusions

We showed that silvestrol is a compound with a potent anti-leukemia activity in *FLT3*-driven AML. These results thereby provide a novel therapeutic strategy in *FLT3*-driven AML by a novel mechanism that is not based on the disruption of the aberrant tyrosine kinase activity through enzymatic TK inhibition, but rather it is effective via translation inhibition of *FLT3* mRNA. Given these and other previously published findings [22,37], silvestrol is now under preclinical development in the National Cancer Institute’s NExT Program for a rapid translation into the clinic.

## Competing interests

The authors declare that they have no competing interests.

## Authors' contributions

HA, DML, MRG and GM designed experiments, critically evaluated the work and took overall supervision in the preparation of manuscript; HA and GM wrote the manuscript; HA performed experiments; RS performed *in vivo* experiments; JGH performed RNA immunoprecipitation; LP and ADK performed silvestrol extraction and preparation for *in vitro* and *in vivo* studies; JJO, CJH, LP, ADK, MAC, DP, JCB, RG provided technical insight and critically reviewed the paper; All authors read and approved the final manuscript.
